# Chronic Disease of the Spinal Cord

**Published:** 1874-01-01

**Authors:** N. S. Davis


### MEDICAL EXAMINER.

A

Semi-Monthly Journal of Medical Sciences.

No. I. CHICAGO, JANUARY 1, 1874. VOL. XV.

### Original Communications.

### CHRONIC DISEASE OF THE SPINAL CORD.

A REPORT PREPARED FOR THE CHICAGO MEDICAL SOCIETY, AT ITS REGULAR MEETING, DEC. 15, 1873. BY N. S. DAVIS, M.D.

ALTHOUGH not a member of this Society, the late Dr. Milton Parker had been a practitioner in this city during the last seventeen years, and was so well known to most of us, that I have thought a brief history of the disease which terminated his life would not be without interest and value.

Dr. Parker was born in 1810, in the State of New Hampshire, and died Dec. 7, 1873, at the age of sixty-three years. His education, both preliminary and medical, was obtained chiefly in Cambridge and Hanover. During the first few years after he entered the profession, he practiced with his father, who was a physician, residing in Acworth, New Hampshire. Desiring the advantages of a milder climate, however, he removed to Virginia in 1845, and continued the practice of his profession in that State until 1856. In the last-named year he became a resident of tnis city, and soon acquired a fair practice, and also won the respect and esteem of a large circle of friends. About three years since he began to complain of a feeling of uneasiness and pressure, accompanied at times by sudden sharp pains in the region of the lower cervical and four upper dorsal vertebræ. There was also tenderness on pressure over the third, fourth and fifth dorsal vertebræ. These symptoms were very variable in the degree of their severity, but seldom were sufficient to interrupt his attention to professional business. After a few months, he found a considerable space over the anterior surface of his chest entirely anæsthetic, or devoid of ordinary sensibility. Then followed, in succession, a great variety of morbid sensations along the spine and in the lower extremeties. These sensations were sometimes like the trickling of cold water, sometimes a prickling heat, and at other times numbness. There were also frequent twitching sensations, like the spasmodic action of individual muscular fibres, more especially in the legs. These symptoms, with the frequent sharp neuralgic pains in the dorsal part of the spine, and sometimes in the legs and feet, were sufficient to cause a great amount of discomfort and loss of sleep, but were not accompanied by any general derangement of digestion, nutrition, and secretion, until within a few weeks before his death. But the steadiness and co-ordination of muscular action in the lower extremities began to fail visibly in the early part of the present year; and such failure steadily increased until, six months since, his gait became so unsteady that it greatly limited his attention to business. The sharp, twinging pains through the back, shoulders, and region of the pectoral muscles, also increased much in frequency and severity.

During the slow progress of his suffering, he received, from time to time, the counsel and advice of several of his medical friends, and submitted to a variety of treatment. He used early and efficient counter-irritation over the affected portion of the spine, and, subsequently, anodyne plasters and liniments. He used, internally, both iodine and mercurial alteratives, the bromides, chloral, ergot, and various tonics, but with very little apparent control over the progress of the disease. About four or five weeks before the fatal result he rather suddenly lost all power of motion in his lower extremities, and, at the same time, suffered a great increase in the pains and muscular twitchings between the scapular and around the chest. To relieve the intensity of his suffering, he called for, and received, from one of his medical friends, daily, a subcutaneous injection of morphia, which afforded much temporary relief. But the paralysis rapidly extended upward until it included the rectum, bladder, and whole lower half of the body. His appetite and digestion failed; hiccough became troublesome; the extremities cool; the pulse feeble; the mucous membrane of the mouth and fauces tender; the mind incoherent; and at last he sank into a pulseless and lethargic condition, which ended in death on the morning of the third day.

Although no *post-mortem* examination was made, there can be no reasonable doubt but that his disease was a slow organic or structural change in the upper half of the dorsal portion of the spinal cord.

Concerning the exact nature of the morbid change in the cord, all might not agree. It differed from cases of ordinary spinal atrophy or progressive locomotor ataxy in these particulars: The pains and local tenderness were both much more severe and persistent; the impairment of muscular motion and co-ordination did not become manifest until a much later period in the progress of the disease than usual; and, in the end, paraplegia came on much more sudden and complete.

My professional familiarity with the case was limited to the last six months of the patient's life, and I regarded it as one of true *sclerosis;* in other words, an inflammatory thickening or hypertrophy of the connective tissue, so interfering with the sensibility and nutrition of the true nerve-matter as to cause both irritation and atrophy, thus accounting for both the unusual pains and progressive failure of sensibility and motion.

